# Patient Satisfaction After Conservative Treatment for Burn Scars in Saudi Arabia

**DOI:** 10.7759/cureus.21896

**Published:** 2022-02-04

**Authors:** Abdulaziz S Almodumeegh, Muhammed R AlKhudair, Abdulaziz F Altammami, Rakan H Alsuhaim, Abdullah I Alhumaidan, Abdulrahman M Alothman

**Affiliations:** 1 Plastic Surgery, College of Medicine, Imam Mohammad Ibn Saud Islamic University, Riyadh, SAU; 2 Medical Student, College of Medicine, Imam Mohammad Ibn Saud Islamic University, Riyadh, SAU

**Keywords:** burn management, skin care, mental health, satisfaction, burn scars, burns

## Abstract

Background

Burn injury is a typical physical injury that occurs as a result of a thermal, electrical, or chemical stimulus. Burn injuries to the skin cause complicated issues, including scar marks, psychological impacts, and affect quality of life (QOL). The preferred treatment technique for burn scars is controversial, as attempts to control the contraction rate remain a challenge, often leading to a poor outcome. Thus, treating burn scar patients is one of the biggest challenges in reconstructive surgery. In this study, we aimed to assess the patients’ perception of pain and QOL after conservative treatment for burn scars in Saudi Arabia.

Methodology

This was a cross-sectional study conducted on Saudi Arabian patients who underwent conservative treatment for their burn scars. Information was obtained using a self-reported questionnaire which was distributed online using Google Forms on several social media platforms. MS Excel was used for data entry, while SPSS version 23 was used for data analysis.

Results

We collected 523 responses to our questionnaire. Among respondents, 70.2% were aged between 18-29 years and 62.1% were single. Most burns were on the upper limbs (74.2%), and 78% of burns were caused by a thermal stimulus (exposure to heat). In terms of emotional status, most respondents did not report significant emotional issues related to their burn scars with a mean emotional score of 12.5 out of 24. Overall, burn scars did not affect the respondents’ ability to work or independence in performing daily work. Regarding the impact of the burns on the patients’ appearance, 29.4% of respondents reported that their scars bothered them significantly, 64.2% reported that their appearance never bothered them, and 11.5% reported that they sometimes tried to forget how their appearance had been changed.

Conclusions

We found that conservative treatment of burn scars is effective in controlling the pain associated with the burns and can improve the patient’s ability to perform work and other daily activities. However, it did not completely improve the psychological distress associated with scars. Further investigations are required to understand the impact of adherence to conservative treatment for burn scars on the psychological status of patients.

## Introduction

Burn injury is a common physical injury that occurs as a result of a thermal, electrical, or chemical stimulus [1]. It is one of the most common medical conditions encountered at emergency departments, accounting for more than 400,000 visits per year in the United States [2]. Burn injuries are also more common in males compared to females, and the home is the most common place of occurrence, followed by the workplace [3].

Burn injuries not only cause permanent scarring, but they may also negatively affect a patient’s psychological health and quality of life (QOL) [4]. Patients with burn scars can experience contractures that limit the range of motion to the affected area and necessitate further interventions to avoid functional limitations [5].

The preferred treatment technique for burn scars is an area of controversy, and poor outcomes remain following attempts to control contraction rates. Thus, the treatment of burn scar patients is a major challenge in reconstructive surgery [6]. Outcome assessments following treatment for burn injuries are now mainly focused on the assessment of the patient’s QOL, including physical and psychological aspects. Mortality and length of hospital stay are less commonly evaluated now, with assessments now focusing on the assessment of functionality and the social problems that patients may face [7].

One of the main indicators of the quality of treatment of burns is the management of pain following burn injury, as burn patients experience persistent acute pain episodes, and thus, the management of pain will significantly influence the patient’s satisfaction. Moreover, pain management can affect many other factors, such as treatment compliance, better clinical outcomes, and the response to treatment [8]. In this study, we aimed to assess patient perception of pain and QOL after conservative treatment for burn scars in Saudi Arabia.

## Materials and methods

This was a cross-sectional study conducted on Saudi Arabian patients who underwent conservative treatment for their burn scars. The study utilized a self-reported questionnaire which was distributed online using Google Forms through several different social media platforms. To obtain reliable results, a sample size of at least 383 patients was determined to be effective for the achievement of a confidence level of 95% (i.e. the real value would be within ±5% of the measured/surveyed value). This was calculated using the calculator.net/sample-size-calculator. The inclusion criteria included: at least one previous burn, conservative treatment for burn scars, Saudi Arabian background, and an age of 20 years or older.

The questionnaire was developed to collect data on sociodemographic characteristics of respondents, including age, marital status, educational level, monthly income, and smoking status. Moreover, the questionnaire collected information on the reported burn, including site of the burn, cause of the burn, degree of burn, time of the burn, and if they needed surgical intervention. We assessed the QOL of respondents after conservative treatment, including assessment of emotional disturbance, scar concerns, impact on work, impact on independence, and impact on general appearance. Each aspect was scored on a scale ranging between 1 and 4, where 1 indicated never and 4 indicated always. For each score, the mean was determined, and the threshold was half of the total possible score where above the threshold reported higher impact. Finally, we assessed the respondents’ perception of pain using three questions about their pain at the time of the study in the last month and the highest pain in the last month. Pain was scored within a range between 1 and 10, where 1 indicated no pain and 10 indicated severe pain. Respondents were also asked to report how the pain felt at the site of the burn.

The study was conducted after respondents provided informed consent for participation approved by the ethical committee at Imam Mohammad Ibn Saud Islamic University. No personal information, including name or address, was requested. MS Excel (Microsoft, Redmond, WA, USA) was used for data entry while SPSS version 23 (IBM Corp., Armonk, NY, USA) was used for data analysis. Frequency and percentage were used for categorical variables while mean and standard deviation were used for description of continuous variables. A Chi test and t-test were used for determining the factors affecting patient satisfaction. P values were considered significant if they were lower than or equal to 0.05.

## Results

We collected 523 responses to the questionnaire. Among those, 70.2% of respondents were aged between 18-29 years, 62.1% were single, and 33.8% were married. A total of 46.3% of respondents indicated having a university degree, while 38% had secondary or lower educational level education. We found that 53.7% of the sample were still students at the time of the study, while 28.2% were employed and 7.6% were not working. In terms of income, 31.7% of the respondents reported a monthly income of between 10000 and 20000 SR, and 17.4% reported receiving less than 5000 SR. Ninety-one percent of the sample were not smoking, while 5.5% were current smokers and 3.1% were former smokers (Table 1).

**Table 1:** Demographic factors of respondents (N=523) SAR: Saudi Riyals

Variable		Count	Column N %
Age (years)	18–29	367	70.2%
	30–39	54	10.3%
	40-49	59	11.3%
	50–59	32	6.1%
	60–69	7	1.3%
	70–79	2	0.4%
	80 and over	2	0.4%
Marital status	Single	325	62.1%
	Married	177	33.8%
	Divorced	11	2.1%
	Widow	10	1.9%
Education level (last educational qualification you obtained)	Secondary or lower education	199	38.0%
	Diploma	37	7.1%
	University	242	46.3%
	Higher education (e.g.: PhD)	45	8.6%
Profession	Employee	148	28.2%
	Student	281	53.7%
	Own work	35	6.9%
	Retired	19	3.6%
	Not working	40	7.6%
Monthly income	<5000 SAR	91	17.4%
	5001–10000 SAR	147	28.1%
	10001–20000 SAR	166	31.7%
	>20000 SAR	119	22.8%
Smoking	No	478	91.4%
	Yes	29	5.5%
	Former smoker	16	3.1%
Duration of smoking	Not smoking	476	91.0%
	<1 year	11	2.1%
	1–5 years	9	1.7%
	6–10 year	12	2.3%
	10–20 years	10	1.9%
	>20 years	5	1.0%

According to questionnaire responses, most burns were on the upper limbs (74.2%), followed by 27.3% on the lower limbs, 10.7% on the face, and 8.8% on the chest and abdomen. Moreover, 78% of burns had a thermal cause (exposure to heat), while sun and chemicals both accounted for 8% of burns. Furthermore, 43% of respondents did not know the degree of their burns, while 32.1% reported first-degree burns, 18.9% reported second-degree burns, and 5.9% reported third-degree burns. Most of the respondents reported that their burn injury had occurred in the last two years (42.6%) and only 3.8% reported the need for surgical intervention (Table 2).

**Table 2:** Characteristics of burns as reported by respondents

Variables		Count	Column N %
Site of the burn	Upper limbs	388	74.2%
	Face	56	10.7%
	Lower limbs	143	27.3%
	Chest and abdomen	46	8.8%
Cause of burn	Thermal (exposure to heat)	408	78.0%
	Electrical	31	5.9%
	Sun	42	8.0%
	Chemicals	42	8.0%
Degree of burn	First degree	168	32.1%
	Second degree	99	18.9%
	Third degree	31	5.9%
	I do not know	225	43.0%
Time of burn	< 2 years	223	42.6%
	2–3 years	128	24.5%
	3–5 year	65	12.4%
	6–10 years	42	8.0%
	>10 years	65	12.4%
Need for surgical intervention	No	503	96.2%
	Yes	20	3.8%

Most respondents did not report significant emotional issues related to their burn scars, and the mean emotional score was 12.5 out of 24. The main emotional issues included no enjoyment in visiting people, being bothered by feelings of loneliness, and feeling sad or depressed (Table 3).

**Table 3:** Emotional status of respondents related to their burn scars 1 = never; 2 = rarely; 3 = mostly; 4 = always

	1	2	3	4	Mean
I often feel sad or depressed	47.0%	25.6%	16.3%	11.1%	1.9
Sometimes, I think I have an emotional problem	48.9%	22.4%	15.9%	12.8%	1.9
I'm bothered by feelings of loneliness	55.6%	19.7%	12.6%	12.0%	1.8
I feel trapped	63.9%	17.8%	10.9%	7.5%	1.6
I don't enjoy visiting people	52.4%	17.6%	15.7%	14.3%	1.9
I don't have anyone to talk to about my problems	60.6%	15.1%	10.1%	14.1%	1.7
I'm not interested in doing things with my friends	67.3%	17.0%	6.7%	9.0%	1.6
Emotional total score	12.5 (out of 28)				

In terms of other concerns about burn scars, 33.3% of respondents hoped that they did not have to do too much to take care of their burns, while 17.4 % reported that taking care of their scars was bothersome. In general, the mean score for burn-associated concerns was 10.05 out of a total score of 20, indicating that burn scars affect patient QOL to a moderate degree. Mostly, burn scars did not affect the respondents’ ability to work. Only 22% reported that they had to stop doing their usual work or duties, and the mean score was 7.1, which was lower than half of the mean of the total score. Moreover, we found that burns did not affect respondents’ independence in terms of doing normal day activities where only 16.8%, 14.1%, and 14.7% of the respondents reported not being able to take a shower independently, to dress themselves, and to get up from a chair, respectively. In terms of the impact of burns on respondents’ appearance, we found that 29.4% reported that their scars bothered them significantly, 64.2% reported that their appearance never bothered them, and 11.5% reported that they sometimes tried to forget how their appearance had been changed. The mean score for general appearance was 7.38, which is lower than half of the average of the total score (Table 4).

**Table 4:** The impact of burn scars on the respondents’ life, work, independence, and general appearance 1 = never; 2 = rarely; 3 = mostly; 4 = always

	1	2	3	4
Taking care of my skin bothers me	51.1%	18.7%	12.8%	17.4%
There are things I've been asked to do to get rid of burns that I don't like to do	56.0%	15.3%	12.6%	16.1%
I hope I don't have to do a lot of things to take care of my burns	39.8%	13.6%	13.4%	33.3%
I find it hard to do all the things I'm told to take care of my burns	53.0%	17.8%	11.5%	17.8%
Taking care of my burns makes it hard to do other things that are important to me	54.9%	20.1%	9.9%	15.1%
Burn-associated concerns	10.05 (Out of 20)			
My burns interfere with my work	77.8%	10.7%	5.4%	6.1%
My burns affected my ability to work	79.2%	10.7%	4.2%	5.9%
My burns caused problems in my work	86.4%	7.3%	3.1%	3.3%
Are you still doing your usual work and duties that you used to do before?	22.0%	5.7%	7.5%	64.8%
Impact on work	7.1 (Out of 16)			
Take a shower independently	16.8%	3.4%	5.5%	74.2%
Dressing yourself	14.1%	3.3%	4.8%	77.8%
Sit and get up from the chair	14.7%	2.7%	4.6%	78.0%
Independence	10.3 (Out of 12)			
My scars are bothering me	43.2%	14.5%	12.8%	29.4%
My general appearance really bothers me	64.2%	15.1%	8.8%	11.9%
Sometimes, I like to forget that my appearance has changed	67.5%	13.2%	7.8%	11.5%
I feel that my burns look unattractive to others	64.2%	9.0%	10.7%	16.1%
General appearance and attractiveness	7.38 (Out of 16)			

**Figure 1:**
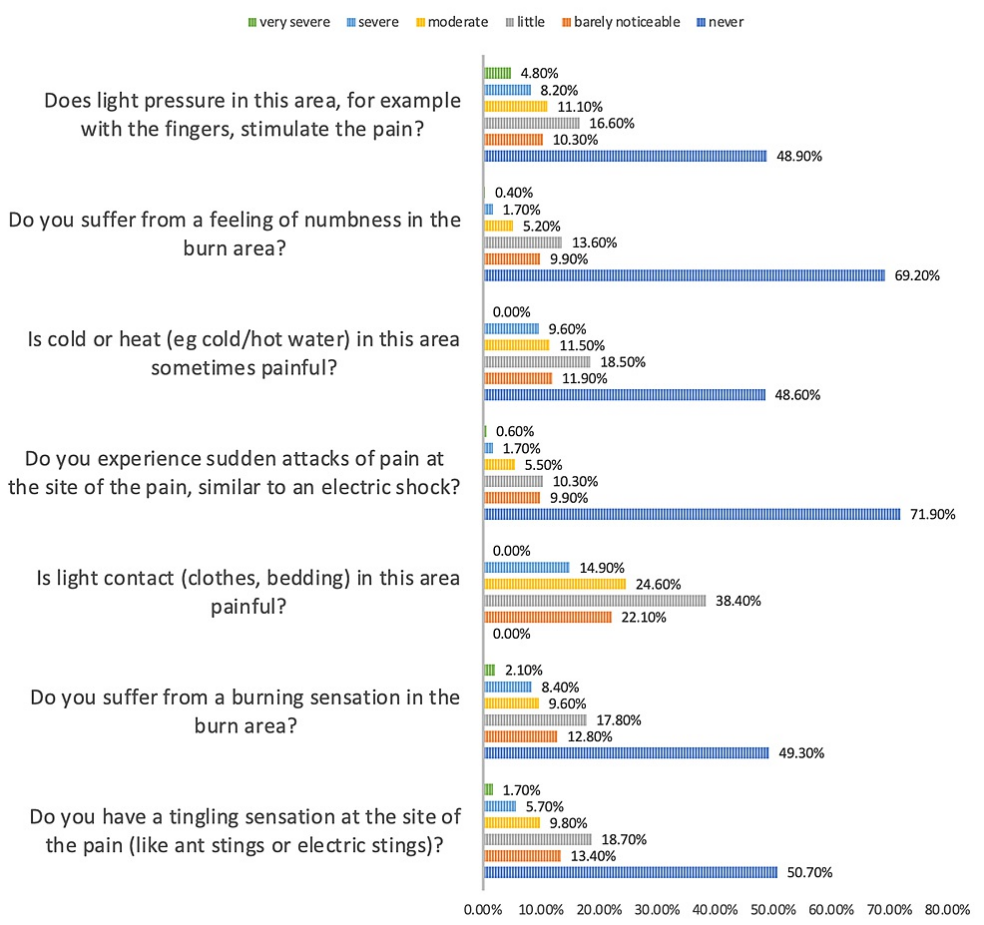
Patients' perception of pain because of burns

Moreover, in terms of the respondents’ perception of pain associated with burns, we found that the mean pain scores at the time of the study, in the last month, and the maximum pain during the last month were 1.3, 2.0, and 3.4, respectively (out of 10), all indicating a low degree of pain. Most respondents (71.9%) had never experienced sudden attacks of pain at the site of the burns, and 69.2% had never suffered from a feeling of numbness in the burn area. However, 51.1% reported that they had some degree of pain after light pressure on the burn area, reaching a severe degree in 4.8% of respondents. All respondents reported some degree of pain when there was light contact with this area. In general, the mean pain score was 7.31 (out of 30) (Figure 1).

Furthermore, we found a significant impact of the cause of the burn on concern about their burns and about their general appearance, with chemical burns associated with greater concerns and less satisfaction with general appearance. In terms of the degree of the burn, we found that second-degree burns were significantly associated with greater concerns, a higher impact on work, and less satisfaction with appearance (P=0.008, 0.025, and 0.000, respectively). Moreover, we found that the need for surgical intervention was associated with a significantly poorer outcome for all aspects (Table 5).

**Table 5:** The relationship between the impact of burn scars on life of respondents and the cause and degree of burns Note: *p<0.05.

		Emotional score	Burn-associated concerns	Impact on work	Independence	General appearance and attractive	Pain range
Cause of burn	Thermal (exposure to heat)	12.71	9.76	7.09	10.31	7.37	7.03
	Electrical	12.35	9.00	7.06	9.32	6.84	6.90
	Sun	10.71	11.74	7.31	10.60	6.52	8.48
	Chemicals	12.88	11.88	7.60	10.55	8.81	9.17
	P-value	0.103	0.001*	0.541	0.308	0.033*	0.210
Degree of burn	First degree	12.10	9.96	7.04	10.65	6.81	6.97
	Second degree	12.55	11.38	7.77	10.55	9.12	8.55
	Third degree	14.00	9.90	7.00	10.00	9.00	8.06
	I do not know	12.67	9.55	6.98	9.96	6.83	6.92
	P-value	0.262	0.008*	0.025*	0.132	0.00*	0.239
Need for surgical intervention	No	12.39	9.86	7.08	10.28	7.24	7.09
	Yes	16.45	14.70	9.00	10.55	11.15	12.90
	P-value	0.00*	0.00*	0.00*	0.710	0.00*	0.00*

## Discussion

Conservative treatments for burn scars, including silicone products and garment compression therapy, have been adopted in high-income countries for over 40 years, although there is little evidence to support their use [9-12]. Non-conservative management includes surgical intervention in treating the affected burnt area. To the best of our knowledge, this is the first study to assess the satisfaction level of Saudi Arabians following conservative treatment for burn scars by assessing their perception of pain and QOL.

In our study, we were able to collect data from 523 respondents with burn scars who had received conservative treatment. Most of our respondents were younger than 39 years old which is similar to other studies which reported that younger people are more likely to suffer burns than older people [13]. Moreover, our analysis showed that the upper limbs were the main organs where burns occurred, followed by the lower limbs, and this result is similar to the results of previous studies [13-16]. The main cause of burns among our population was thermal (including exposure to heat) and this result was similar to the results of Al-Qahtani et al. [13] and the results of a cross-sectional study conducted by Agbenorku et al. in Ghana which suggested that scalding or thermal burns were the main causes of burns, especially among younger participants [17]. A systematic review conducted by Bombaro et al. reported the same results [12]. Furthermore, we found that most of the respondents did not know the degree of their burns, which indicated that most burns were superficial and not severe, which is similar to other studies that reported that most burns are not severe [12,13,18]. Moreover, only 3.8% of respondents reported that they needed surgical intervention for their burns.

We found that burns scars had a negative impact on the respondents’ emotional status, including reports of not enjoying visiting people, being bothered by feelings of loneliness, and feeling sad or depressed, even after conservative treatment. According to Van Leoy, living with scars in a society that places high value on beauty can be challenging for a burn survivor; therefore, psychological problems may occur in burn survivors with visible scars [19]. In our study, most burns were on the upper limbs, and therefore quite visible, and this could explain the poor psychological status of our study population. Many studies have elaborated on the psychological, physical, and social consequences in burn-affected patients [20], with the main reasons cited being the changes in body image and their negative perception being socially unaccepted [21]. Most burn survivors exhibit high rates of depression. In our study, many respondents reported that their scars were bothering them; however, they were not bothered by their appearance.

In terms of the presence of burn scars on a respondent’s ability to work, we found that most respondents reported that their scars did not affect their ability to work, or to perform other daily activities such as taking a shower or putting clothes on, indicating the effectiveness of conservative treatment for scars or the mild condition of the scars. This result is similar to that of previous studies [13].

The degree of pain associated with burns is considered one of the important factors relating to psychological disorders [13,15,17]. We found that the pain score was reduced after conservative treatment, with a pain score of 1.3 (out of 10) at the end of the treatment period compared to 2.0 (out of 10) at the beginning of treatment and that most respondents did not feel any itching or pain at burn sites, except with contact. This indicated the effectiveness of conservative treatment in controlling the pain associated with the burns.

This study had some limitations, including its dependence on a self-reported questionnaire which may suggest the inclusion of some personal bias leading to some respondents not providing honest answers on the questionnaire, as well as an inability to report their degree of burns accurately. Moreover, the questionnaire was distributed online which may have created some sampling bias toward younger respondents. Finally, some questions required feedback about information from the past and this may have led to recall bias.

## Conclusions

In conclusion, we found that conservative treatment of burn scars is effective in controlling the pain associated with burns and improving the patients’ ability to work and perform other daily activities. However, it did not completely improve the psychological distress associated with scars. Further investigations are required to understand the impact of adherence to conservative treatment for burn scars on the psychological status of patients.

## References

[1] Peck MD (2011). Epidemiology of burns throughout the world. Part I: Distribution and risk factors. Burns.

[2] (2022). National Hospital Ambulatory Medical Care Survey: 2013 Emergency Department Study Tables. https://www.cdc.gov/nchs/data/ahcd/nhamcs_emergency/2013_ed_web_tables.pdf.

[3] American Burn Association (2022). Burn Incidence Fact Sheet. ABA National Burn Repository.

[4] Mazharinia N, Aghaei S, Shayan Z (2007). Dermatology Life Quality Index (DLQI) scores in burn victims after revival. J Burn Care Res.

[5] Schouten HJ, Nieuwenhuis MK, van Zuijlen PP (2012). A review on static splinting therapy to prevent burn scar contracture: do clinical and experimental data warrant its clinical application?. Burns.

[6] Stekelenburg CM, Marck RE, Tuinebreijer WE, de Vet HC, Ogawa R, van Zuijlen PP (2015). A systematic review on burn scar contracture treatment: searching for evidence. J Burn Care Res.

[7] Oh H, Boo S (2017). Assessment of burn-specific health-related quality of life and patient scar status following burn. Burns.

[8] Browne AL, Andrews R, Schug SA, Wood F (2011). Persistent pain outcomes and patient satisfaction with pain management after burn injury. Clin J Pain.

[9] Friedstat JS, Hultman CS (2014). Hypertrophic burn scar management: what does the evidence show? A systematic review of randomized controlled trials. Ann Plast Surg.

[10] Atiyeh BS, El Khatib AM, Dibo SA (2013). Pressure garment therapy (PGT) of burn scars: evidence-based efficacy. Ann Burns Fire Disasters.

[11] Engrav LH, Heimbach DM, Rivara FP (2010). 12-Year within-wound study of the effectiveness of custom pressure garment therapy. Burns.

[12] Bombaro KM, Engrav LH, Carrougher GJ (2003). What is the prevalence of hypertrophic scarring following burns?. Burns.

[13] Almutlaq BA, Jarman A, Alfraihi R (2020). Skin burns in Saudi Arabia: Causes, management, outcomes and quality of life after skin burns. Int J Burns Trauma.

[14] Ai JW, Liu JT, Pei SD, Liu Y, Li DS, Lin HM, Pei B (2017). The effectiveness of pressure therapy (15-25 mmHg) for hypertrophic burn scars: A systematic review and meta-analysis. Sci Rep.

[15] Wiseman J, Simons M, Kimble R, Ware R, McPhail S, Tyack Z (2017). Effectiveness of topical silicone gel and pressure garment therapy for burn scar prevention and management in children: study protocol for a randomised controlled trial. Trials.

[16] Hultman CS, Edkins RE, Lee CN, Calvert CT, Cairns BA (2012). Shine on: Review of laser- and light-based therapies for the treatment of burn scars. Dermatol Res Pract.

[17] Agbenorku P, Akpaloo J, Farhat BF (2010). Burn disasters in the middle belt of Ghana from 2007 to 2008 and their consequences. Burns.

[18] Ghoddusi Johari M, Mohammadi AA, Dastgerdi V (2019). Burn: A predictable but preventable tragedy in epileptic patients. World J Plast Surg.

[19] (2020). Van Loey NEE: Psychological Impact of Living with Scars Following Burn Injury. Textbook on Scar Management.

[20] Van Loey NE., Faber A., Taal L (2001). Do burn patients need burn specific multidisciplinary outpatient aftercare: research results. Burns.

[21] Lawrence JW, Rosenberg LE, Fauerbach JA (2007). Comparing the body esteem of pediatric survivors of burn injury with the body esteem of an age-matched comparison group without burns. Rehabil Psychol.

